# Supplementary material for: Splice-Junction-Based Mapping of Alternative Isoforms in the Human Proteome
Source: Cell Rep. Author manuscript; Available in PMC 2020 Jan 15. (PMC6961840; doi:10.1016/j.celrep.2019.11.026)
Supplement: 2 [file NIHMS1546469-supplement-2.zip › DF1/PXD006675/PulmonaryVein/PulmonaryVein_23_CLTCL1_LQEHFQLQNLGINPANIGFSTLTMESDK.pdf]

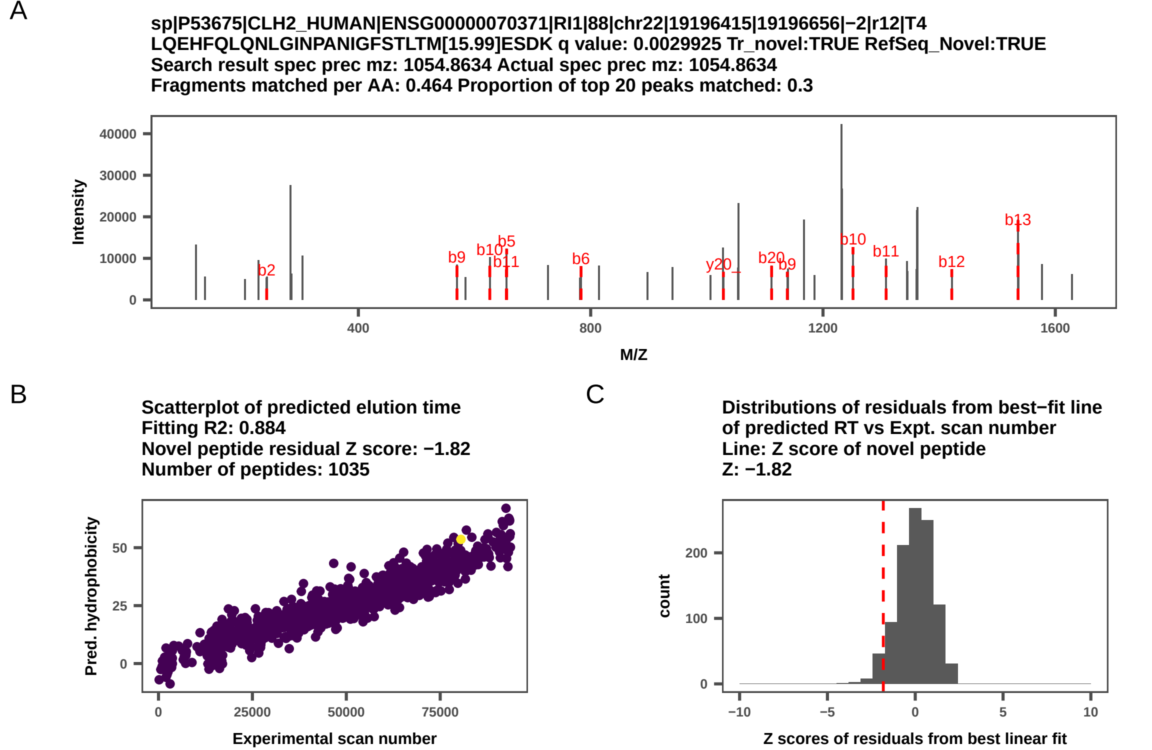
A
sp|P53675|CLH2_HUMAN|ENSG00000070371|RI1|88|chr22|19196415|19196656|−2|r12|T4
LQEHFQLQNLGINPANIGFSTLTM[15.99]ESDK q value: 0.0029925 Tr_novel:TRUE RefSeq_Novel:TRUE
Search result spec prec mz: 1054.8634 Actual spec prec mz: 1054.8634
Fragments matched per AA: 0.464 Proportion of top 20 peaks matched: 0.3
Intensity
40000
30000
20000
10000
0
b2
b9
b10
b5
b11
b6
y20
b20
b9
b10
b11
b12
b13
400
800
1200
1600
M/Z
B
Scatterplot of predicted elution time
Fitting R2: 0.884
Novel peptide residual Z score: −1.82
Number of peptides: 1035
Pred. hydrophobicity
50
25
0
0
25000
50000
75000
Experimental scan number
C
Distributions of residuals from best−fit line
of predicted RT vs Expt. scan number
Line: Z score of novel peptide
Z: −1.82
count
200
100
0
−10
−5
0
5
10
Z scores of residuals from best linear fit
